# Reflections: enhancing critical thinking in science education by implementing philosophy elements into training

**DOI:** 10.1128/msphere.00399-24

**Published:** 2024-07-02

**Authors:** Ilinca I. Ciubotariu

**Affiliations:** 1 Department of Molecular Microbiology and Immunology, Bloomberg School of Public Health, Johns Hopkins University, Baltimore, Maryland, USA; University of Michigan, Ann Arbor, Michigan, USA

**Keywords:** philosophy, science, education, interdisciplinary

## Abstract

In this work, I describe the trajectory of philosophy and science as separate disciplines from their early days as quite overlapping fields to their clear divergence in the latest centuries. From personal experience, I discuss the benefits of exposure to philosophy and closely related courses in undergraduate studies and bring to the forefront the positive aspects of integrating philosophy of science courses in graduate science, technology, engineering, and mathematics (STEM) curriculum. I also briefly offer some additional steps institutions can take to foster unity between areas of science and philosophy by incorporating interdisciplinary activities.

## INTRODUCTION

The origins of philosophy and science date back to the times of ancient civilizations, where thinkers sought to understand the world and its underlying principles. Although it can be argued that philosophers such as Pythagoras, Democritus, and Socrates did not directly engage in scientific investigations, they all incorporated elements of science into their respective inquiries. They defined the concept of a harmonious and orderly universe governed by mathematical principles, proposed the atomic theory, and devised a form of argumentative dialogue between individuals with an emphasis on posing questions, respectively, and many of these works concomitantly laid the groundwork for elements of modern science, such as logical reasoning, empiricism, reductionism, and ethical and moral considerations. Similarly, in the inquiries of greats such as Aristotle or Plato, we can appreciate that their explorations of the natural world resembled present-day scientific investigations.

Hence, if we reflect to many parts of history, we are quickly reminded that philosophy was the mother of all sciences (1) (or sometimes referred to as “queen” [2]) and focused on the study of the nature of the world, understanding, and developing reason. Additionally, for the ancient Greeks, “science” and “philosophy” were normally used interchangeably to describe investigations ranging from the senses to abstract principles (3). Moreover, “science,” or, as it was then often referred to, “natural philosophy,” or the study of physics (nature), was intimately associated with the field of philosophy for hundreds of years.

Fast forward a few centuries into the 17th century, we have a time that was marked by the Scientific Revolution, with figures such as Francis Bacon, René Descartes, Isaac Newton, and Galileo Galilei bringing to light the scientific method with great emphasis on empiricism and experimentation. Moreover, this period marked “the birth of modern science and philosophy” (3). At this point, the understanding of the structure and roles of philosophy and science were well described by the philosopher and mathematician Descartes (4, 5): “*toute la philosophie est comme un arbre, dont les racines sont la métaphysique, le tronc est la physique, et les branches qui sortent de ce tronc sont toutes les autres sciences, qui se réduisent à trois principales, à savoir la médecine, la mécanique et la morale”* (translation: “all philosophy is like a tree, whose roots are metaphysics, the trunk is physics, and the branches that come out of this trunk are all the other sciences, which are reduced to three main ones, namely medicine, mechanics and morality”). In other words, he likens philosophy to a tree and indicates that its roots are formed by metaphysics, the trunk supported by the roots is physics, and the branches that emanate from the trunk are all the other sciences: namely, medicine, mechanics, and morality. In this sense, the metaphorical tree holds all the stages of unified knowledge (3) and one can interpret that philosophy is not an isolated discipline, but rather one that is fundamentally connected to all forms of human inquiry and knowledge.

This Cartesian model of philosophy stuck around for many years, and many discussions emerged along the way, including *Gnoseologie* or *Erkenntnistheorie* (philosophic theory of knowledge or enlightenment)/epistemology, philosophy of science, analytical philosophy, *Wissenschaftslehre* (Fichte’s science of knowing), and *Naturwissenschaften* (the science of nature), etc. (6). However, following this gradual and long period of growth, around the late 18th century and the beginning of the 19th century, the demarcation between science and philosophy started to become visible, some even referring to the “divorce” between scientists and philosophers, who each began to view their respective fields as separate entities (3, 7). For instance, the gap between philosophy and science was expressed by Immanuel Kant, who strongly described their separate entities as “science proper” and “philosophy proper” (3). Similarly, in the early 1800s, French philosopher Théodore Simon Jouffroy claimed that philosophy consists of the problems that science has not solved (8, 9): *“Qu'est-ce donc que la philosophie? C'est la science de ce qui n'a pas encore pu devenir l'objet d'une science*” (translation: “What then is philosophy? It is the science of that which has not yet been able to become the object of a science”) (10). This encapsulated a common perspective on the relationship between philosophy and science, which suggested that philosophy occupies the intellectual terrain that science has not yet explained or even more so, cannot explain due to its inherent methodological or empirical limitations.

Although this piece will not go deeper into the intricacies of the development of science or philosophy throughout history to modern day, it is undeniable that science and philosophy shared an interconnected history (11). Around the late 18th century, scientific methodologies diverged from philosophical inquiry, with science developing distinct epistemology with a focus on systematic observation, verifiable evidence, and rigorous methodology, whereas philosophy continued to explore broader questions with a focus on existence, conceptual analysis, and metaphysics. The field of science gained its modern shape, with the emergence of disciplines such as biology, astronomy, physics, and so on. Since then, the gap between science and philosophy has mostly remained in practice today, with the two fields often taught separately in universities as if they represent two opposed modes of thinking (8), highlighting the complex relationship between science and philosophy in modern day.

## COMPLEX RELATIONSHIP BETWEEN SCIENCE AND PHILOSOPHY

For instance, many may be familiar with the anonymous words (sometimes attributed to physicist Richard Feynman), “Philosophy of science is about as useful to scientists as ornithology is to birds” (12), evidently critiquing the practical impact of philosophers of science to science. By drawing on ornithology and birds themselves, the comparison aims to highlight a distinction between theoretical understanding and practical utility. Essentially, considering a practical approach to science, these words could be interpreted to emphasize the empirical basis of science, possibly finding philosophy’s abstract nature less applicable to scientific activities. Moreover, while philosophy examines science, it might not directly contribute to scientific practice, a point often debated among scientists and philosophers alike. This interpretation posits that scientists, like birds, do not fundamentally need the philosophy of science to conduct experiments or make scientific discoveries.

However, I argue that just as ornithology might serve a purpose to birds, philosophy of science can contribute to a deeper understanding of scientific methods, the nature of scientific reasoning, and the implications of scientific discoveries. Two recent works mused on this topic (13, 14) and argued that the field of science does need philosophy, and the discipline of medicine would also benefit from the interdisciplinarity. Laplane et al. (14) supported their claim by providing examples of how philosophy can contribute to the advancement of science, such as through conceptual clarifications and criticism of scientific assumptions. Andreoletti et al. (13) similarly argued that these findings could be extended to medicine and even suggested for a course of philosophy of science to be integrated into the curriculum for medical students, with special topics on statistical concepts. Moreover, others have called for educating microbiologists in the process of thinking (15).

Indeed, philosophy can provide a critical framework for scientists to question the foundations and ethical dimensions of their work, thereby fostering a more reflective and responsible approach to science. Along the same thread of thought, in a recent piece titled “The call of science” (16), Robert Pennock astutely wrote that this metaphor did not have to be discouraging; in fact, “philosophy of science can delineate the features of the scientific mindset so that these might be better understood and improved to advance a flourishing research culture.” I believe that the philosophy of science plays a significant role in analyzing the foundations, assumptions, and implications of scientific methods and practices. It helps contextualize science within broader intellectual and social frameworks, guiding ethical considerations and theoretical developments.

Further, let us look at the opposite side of the coin and use the words of the mathematician and philosopher Alfred North Whitehead, which succinctly emphasize the drawbacks of the separation of philosophy and science: “the antagonism between philosophy and natural science has produced unfortunate limitations of thought on both sides. Philosophy has ceased to claim its proper generality, and natural science is content with the narrow round of its methods” (17). One common critique of PhD programs in science, technology, engineering, and mathematics (STEM) and beyond is that they train students so narrowly, and as students become specialized in one specific subtopic (18), they might not develop the ability to think broadly and critically. Thus, the question that emerges is how can we balance the integration of philosophical in science disciplines and further encourage interdisciplinarity?

There is a lot of interconnectedness when it comes to philosophy and science that is fundamentally important, from which scientists-in-training could benefit. Although science and philosophy are not the same and we may often ask ourselves “is philosophy a science?” (19), they are indeed “closely related, so that it is sometimes hard to say how far a man is a scientist, and how far a philosopher” (20). Moreover, perhaps philosophy could also benefit from rigorous science. After all, Edmund Husserl aimed to ground philosophy in scientific rigor by developing phenomenology to systematically analyze consciousness and the structures of experience (6, 21–23). Certainly, philosophers are not always scientists (8) and vice versa, and that is not the goal, so we can instead advocate for adapting principles inherent to philosophical thinking when conducting our work. Herein, I describe some personal musings of incorporating philosophy training to create more critical thinkers in science disciplines.

## REFLECTIONS FROM PERSONAL EXPERIENCE

On reflection, the core humanities curriculum I was exposed to during my undergraduate education expanded my critical thinking and appreciation for in-depth investigation. I resonate well with the reflection of the theoretical physicist Werner Heisenberg and 1932 Nobel Prize winner known for his uncertainty principle and work on nuclear fission, on his humanities training: “My mind was formed by studying philosophy, Plato and that sort of thing.” By expressing that his intellectual foundation was molded by studying philosophy, he emphasizes the underlying power of philosophy to expand the mind and offers support for training in logical thinking and rational inquiry. In fact some have argued that “many great scientific innovators have at some point studied the works of philosophers and developed philosophical views of their own” (24), with philosophy playing a heuristic role in the discovery of scientific theories (25).

I, too, can argue that I have (and perhaps unknowingly at the time) benefited from the integration of philosophy as complementary studies to my STEM pursuits. In my undergraduate curriculum, philosophy courses led me to dissect works such as those by Jean-Jacques Rousseau, René Descartes, and David Hume. In retrospect, the work of René Descartes in which he outlines his approach for understanding the natural world and uncovering truth through reason and skepticism is particularly relevant to science education. He discussed four precepts: doubting everything, dissecting problems into smaller parts, solving simpler questions first, and being thorough, all of which formed the basis of deduction. These steps comprise reasoning and rationalism, which is a large portion of the scientific process, with the others being experimentation and inductive reasoning. This can enable one to combine both logic and observation in one’s research. Similarly, in my French and Romanian literature courses, I dissected works such as *Le Père Goriot* by Honoré de Balzac and drew connections between his process of writing and the methods of science: namely, he transposed the natural sciences into reality through his approach of including specific details. This exercise is key for reproducibility today, as sufficient details are needed for different individuals to replicate experiments and achieve consistent results. I unpacked Jacques Derrida’s *Che Cós'è la Poesia?*, in which he tried to eliminate the gap between what he wrote about and what his writing was effectively doing. These two elements are critical for scientific communication at all levels, especially with respect to the necessity of scientific advice being clear and concise. When reading Lucian Blaga’s *Trilogy of Values*, I realized he advocated for investigating the philosophical and cultural infrastructure of scientific theories. This work can push us to examine ethics and gain an appreciation of placing one’s work in context of societal implications; for instance, in my own research, it moved me to think about the consequences different types of malaria control might have on local communities.

Taking these personal connections I drew between philosophy and science further on my pursuit of advanced science education, I stumbled upon a novel, innovative graduate program that aims to bridge science and philosophy and emphasize their interconnected nature: housed in the R³ Center for Innovation in Science Education (R3ISE; https://publichealth.jhu.edu/the-r3-center-for-innovation-in-science-education), within the Department of Molecular Microbiology & Immunology at the Johns Hopkins Bloomberg School of Public Health, the R3 Program (https://publichealth.jhu.edu/the-r3-center-for-innovation-in-science-education/r3-programs-at-the-bloomberg-school-and-johns-hopkins-university) stands for the three fundamental “R” principles of good scientific practice: rigorous research, reproducibility of scientific work, and responsibility of science practitioners to society. It comprises courses that embrace and emphasize these core values while shaping the next generation of big-picture thinkers with strong critical thinking skills (26). This program aims to “put the ‘Ph’ back in PhD” (27), and other institutions are already adopting the R3 model for training future health practitioners to be broader thinkers, not just specialists (28).

As part of my graduate education in the Department of Molecular Microbiology & Immunology, I enrolled in these foundational courses, which covered works by Aristotle, Isaac Newton, Karl Popper, Thomas Kuhn, among others, in the context of their transformational impact on science inquiry. Students have the opportunity to explore the philosophical foundations of science, effective communication, and practical ethics; ask pertinent questions, such as what science itself is and where its limits fall; define the differences between science and truth and belief; and discuss the nature of scientific errors and their avoidance in practice. As such, the R3 courses nurtured multiple skills ranging from technical research design to creative and ethical reasoning to problem-solving.

It was during this program that I truly began to see the intersections of philosophy, as if the sections of a Venn diagram blurred together. I started to formulate questions to ask about processes and focus more on the “how” and “why” elements of science, rather than “what.” I also learned the importance of understanding the impact and consequences of one’s body of work to human society. It was through these courses with strong philosophical roots that I strengthened my training as an inquisitive scientist and developed the ability to raise critical questions, hypotheses, and logical connections—all of which form the basis of science inquiry. I would highly recommend other graduate training programs to incorporate such courses into their training of future generation of scientists. Similarly, like Albert Einstein advocated (25), I would also argue that there is great impact of history and philosophy of science training on scientific investigations as it can provide scientists with independence of thought and consequently broaden their mindsets with critical thinking.

## OTHER POSSIBLE STEPS

Additionally, below are a few, basic recommendations to add to suggestions offered by Laplane et al. for graduate programs as complementary measures to implementing philosophy and ethics courses in their training curriculum.

- Interdisciplinary departmental seminars: organize seminars where graduate students from both science and humanities-focused programs can come together to discuss common topics, encouraging cross-disciplinary dialogue and collaboration
- Philosophy sections at STEM conferences: introduce specific sections at STEM conferences dedicated to exploring philosophical elements as it applies to subtopics, such as microbiology, infectious diseases, etc.
- Cross-disciplinary grant offerings for STEM and humanities: as an institution, offer calls for grant submissions that will involve investigators from a STEM discipline and humanities subject to build bridges between the fields
- Philosophy in lab meetings: dedicate a few lab meeting sessions to incorporate philosophical discussions, where graduate students can explore ethical, epistemological, and methodological implications of their research
- Virtual reality philosophy labs: develop environments where graduate students can explore thought experiments in immersive, interactive simulations, thus bridging philosophical inquiry with cutting-edge technology

I end by echoing the words of Claude Bernard (29): *“et il conclut: cette union solide de la science et de la philosophie est utile aux deux, elle élève l'une et contient l'autre. Mais si le lien qui unit la philosophie à la science vient à se briser, la philosophie, privée de l'appui ou du contrepoids de la science, monte à perte de vue et s'égare dans les nuages, tandis que la science, restée sans direction et sans aspiration élevée, tombe, s'arrête ou vogue à l'aventure.”* (Translation: “and he concludes: this solid union of science and philosophy is useful to both, it elevates one and contains the other. But if the link which unites philosophy to science becomes broken, philosophy, deprived of the support or counterweight of science, rises as far as the eye can see and gets lost in the clouds, while science, remaining without direction and without high aspiration, falls, stops, or sails on adventure.”) These words underscore the importance of maintaining exchange between science and philosophy to foster intellectual advancement in the continued pursuit of knowledge. When science and philosophy are closely connected, both disciplines can benefit from each other, and when the link is severed, both disciplines might lose their way; hence, we should continue incorporating philosophy into science training, especially at the graduate and even undergraduate levels.
